# Antibacterial and Cytotoxic Effects of *Moringa oleifera* (Moringa) and *Azadirachta indica* (Neem) Methanolic Extracts against Strains of *Enterococcus faecalis*

**DOI:** 10.1155/2018/1071676

**Published:** 2018-09-25

**Authors:** Lucía Arévalo-Híjar, Miguel Ángel Aguilar-Luis, Stefany Caballero-García, Néstor Gonzáles-Soto, Juana Del Valle-Mendoza

**Affiliations:** ^1^School of Odontology, Health Sciences Faculty, Universidad Peruana de Ciencias Aplicadas-UPC, Lima, Peru; ^2^Research Center and Innovation of the Faculty of Health Sciences, Universidad Peruana de Ciencias Aplicadas, Av. San Marcos cdra 2, Cedros de Villa, Lima, Peru; ^3^Institute of Nutritional Research (IIN), 1885 La Molina Ave., Lima 12, Peru

## Abstract

**Objective:**

To evaluate antibacterial and cytotoxic effect of 2 methanolic extracts of *Azadirachta indica* and *Moringa oleifera* against strains of *Enterococcus faecalis* (ATCC 29212) *in vitro*.

**Methods:**

The methanolic extracts of *Azadirachta indica* and *Moringa oleifera* were prepared *in vitro*. The antibacterial effect of the extracts against *Enterococcus faecalis* was evaluated using the agar diffusion technique. The minimum inhibitory concentration (MIC) was determined using the microdilution method and the cytotoxicity using the cellular line MDCK.

**Results:**

The methanolic extract with the most antibacterial effect during the first 24 and 48 hours against *Enterococcus faecalis* was *Moringa oleifera*, evidencing a growth inhibition zone of 35.5 ± 1.05 and 44.83 ± 0.98, respectively. The MIC for both extracts was 75 *µ*g/ml. The bactericidal effect of the *Azadirachta indica* extract was found at a concentration of 25 *µ*g/ml and a concentration of 75 *µ*g/ml for *Moringa* extract.

**Conclusions:**

In conclusion, we demonstrated that the methanolic extract of *Azadirachta indica* and *Moringa oleifera* both have an antibacterial effect against *Enterococcus faecalis* strains during the first 24 and 48 hours. None of the extracts exhibited toxicity against the cell lines under low concentrations.

## 1. Introduction

One of the main objectives of endodontics is root canal disinfection, mainly associated with the colonization of anaerobic bacteria. There are endodontic treatments that fail due to a complex root canal anatomy or antibiotic resistance of some bacterial families, with *Enterococcus faecalis* being the most resistant bacteria reported [1–6].

Various antibacterial agents have been previously studied, and they can be obtained from natural sources or synthetic agents [7]. Furthermore, recent studies have reported the pharmacological properties and high medicinal value of a myriad of natural extracts. Some of these products, however, have been used empirically, leaving aside the study and determination of its properties [8].

The contemporary field of phytotherapy focuses on the study of plants and its pharmacological properties to treat diseases. *Azadirachta indica* also known as neem and *Moringa oleifera* also known as moringa are both native Indian trees known for their high medicinal values due to their curative properties [9–15]. *A. indica* has analgesic, antifungal, and antibacterial properties and has therefore been used as treatment for gastrointestinal ailments, mouth hygiene, and certain chronic diseases such as diabetes, high blood pressure, and dyslipidemia [16–19]. On the other hand, *M. oleifera* has been reported to have antiviral, antioxidant, antisclerotic, antibacterial, and anti-inflammatory properties. It has been used as treatment for malaria, malnutrition, colon cancer, and myeloma [20].

Even though these plants have been studied to treat the previously mentioned ailments, no research evaluates if time is directly correlated with the antibacterial effect of *A. indica* against *E. faecalis*. On the other hand, there has yet to be any research on the antibacterial effect of *M. oleifera* or its cytotoxicity as a root canal disinfectant.

The objective of this study was to evaluate the antibacterial and cytotoxic properties of methanolic extracts of *A. indica* and *M. oleifera* against some *Enterococcus faecalis* strains (ATCC 29212) *in vitro*.

## 2. Materials and Methods

### 2.1. Sample

The sample size was determined with a formula of comparative means using the statistical software Stata® version 12.0. A confidence interval of 95% was considered, and a power of 80% was used. The mean and standard deviation parameters for a group were obtained using a pilot test that was previously run. Finally, a sample of 6 wells per group was established.

### 2.2. Extract Preparation

Fresh *A. indica* leafs and pulverized *M. oleifera* leafs were obtained from a naturist shop. Both were free of impurities and had a sanitary registry. These products were placed in different containers and were treated with absolute methanol (1 : 2 weight/volume) to be later stored at room temperature for 10 days without sunlight exposure. The solutions were filtered through Whatman paper no. 4 and placed in labeled tubes. A rotatory evaporator was then used at 50°C to separate the methanol by distillation to finally obtain a pure extract. The extracts were stored at 4°C until use.

### 2.3. Bacterial Cultures

*Enterococcus faecalis* ATCC 29212 was obtained from GenLab Laboratory in Peru, a representative of MicroBiologics Laboratory (USA). The bacteria were cultured in agar BHI (brain heart infusion) in anaerobic conditions at 37°C for 72 hours. 3 to 4 colonies were then isolated and later inoculated in 3 mL of a BHI broth under the same conditions previously mentioned. The cultures were then diluted in a sterile saline solution to reach a McFarland scale density of 0.5, which approximately estimates a concentration of 1.5 × 10^8^ CFU/mL.

### 2.4. Antimicrobial Activity Evaluation

Antimicrobial activity evaluation was achieved using the well diffusion method [21]. BHI agar was prepared and autoclaved at 121°C for 15 minutes. The agar was left to cool, and then the previously prepared bacterial suspension was inoculated. Finally, we proceeded to add the agar to sterile petri dishes. A sterile cork borer was used to punch 9 mm holes on the agar plate which were then filled with 1000 mL of each methanolic extract. In addition, 2% chlorhexidine was used as the positive control and 1× saline solution as the negative control. The agar plates were incubated at 37°C, and the diameters of the inhibition growth zones were measured during the first 24 and 48 hours in millimeters with a vernier caliper.

### 2.5. Determination of the Minimum Inhibitory Concentration (MIC)

The minimum inhibitory concentration was determined using the microdilution method described by Gupta and Negi [22]. Each methanolic extract underwent serial dilutions (1/2), and each solution was added to the wells in the petri dishes with concentrations ranging from 1.56 to 75 *µ*g/ml. In addition, 2% chlorhexidine was used as the positive control and 1× saline solution as the negative control. The plates were incubated under anaerobic conditions at 37°C for 24 hours. The minimum inhibitory concentration was considered as the minimum concentration of the extract that inhibited bacterial growth.

### 2.6. Determination of the Minimum Bactericidal Concentration (MBC)

The methanolic extract dilutions were added into each well of a 96-well microtiter plate, and then the bacterial suspension was inoculated. The plate was incubated at 37°C for 24 hours. In order to determine the minimum bactericidal concentration (MBC), aliquots were pipetted out of each well and seeded onto the agar. The sample petri dishes were incubated under anaerobic conditions at 37°C for 24 hours. The minimum bactericidal concentration is defined as the minimum concentration required to inhibit any bacterial growth.

### 2.7. Cytotoxicity Evaluation

Cytotoxicity was evaluated by means of a colorimetric assay based on the reduction of MTT by mitochondrial enzymes [23]. A 96-well microtiter plate was used to culture 1 × 10^4^ cells per well. The plate was then incubated at 37°C in a humid atmosphere of 5% CO_2_ for 24 hours. Subsequently, we proceeded to pipette the methanolic extracts *Azadirachta indica* (neem) and *M. oleifera* (moringa) with concentrations ranging from 0 to 100 *µ*g/mL onto the monocellular layer. Each concentration assay had a positive control for cellular viability (culture medium without any extracts). The plates were incubated at 37°C for 6 days, and the morphology of the cells was surveilled daily. The cytotoxic concentration 50 (CC_50_) is defined as the concentration of a substance required to decrease cellular viability by 50%.

Twenty microliters of MTT solution (3 mg/ml in PBS 1X) was added into each well and then left incubating for 3 hours. The medium was carefully removed to obtain the formazan crystals which were then diluted with 200 *µ*L of DMSO (dimethyl sulfoxide). In order to determine cellular viability, a microplate photometer was used to measure and compare the absorbance of the treated cultures against the nontreated culture. The results were analysed with the computer software Pharm/PCS. The monolayers were observed under the microscope to evaluate any morphological changes.

### 2.8. Statistical Analysis

The antibacterial effect was registered in millimeters (mm). It was analysed by means of a nonparametric test, Kruskal–Wallis, with a statistical significance level of 5% for the comparison between *A. indica*, *M. oleifera*, and chlorhexidine. Data were analysed using the statistical package Stata® version 12.0.

## 3. Results

### 3.1. Antimicrobial Activity of the Natural Extracts

After the first 24 hours, the methanolic *A. indica* extract generated a growth inhibition zone of 33.16 mm and the extract of *M. oleifera* generated a 35.5 mm zone. At 48 hours, the growth inhibition zones were 38.83 mm and 44.83 mm, respectively. The methanolic extract of *M. oleifera* had a greater antibacterial effect in comparison with chlorhexidine (control group) and the methanolic extract of *A. indica*. Furthermore, statistically significant differences were found for both groups at 24 and 48 hours (*p* < 0.01) (Table 1).

### 3.2. MIC, MBC, and Bacteriostatic Effect of *A. indica* and *M. oleifera* Extracts

The MIC of both the methanolic extracts, *A. indica* and *M. oleifera*, was 75 *µ*g/ml, demonstrating a bacteriostatic concentration up to this value.

Additionally, the bactericidal concentrations for *A*. *indica* and *M*. *oleifera* were 25 *µ*g/ml and 75 *µ*g/ml, respectively (Table 2).

### 3.3. Cytotoxicity of the Methanolic Extracts of *A. indica* and *M. oleifera*

**Figure 1:**
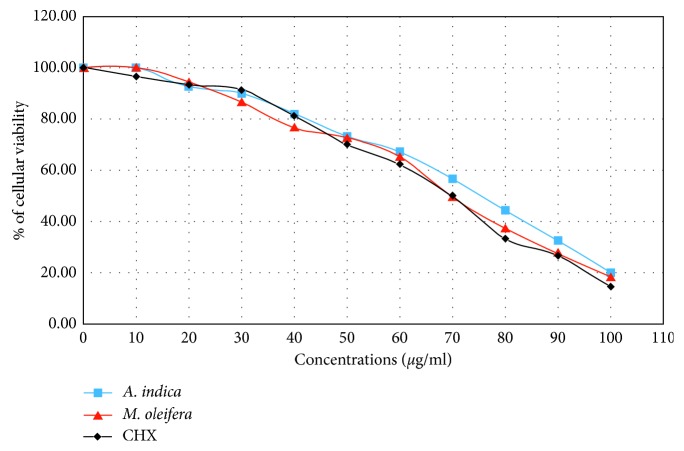
Cellular viability evaluation of Jurkat cells against the methanolic extracts of *Moringa oleifera* and *Azadirachta indica* against *Enterococcus faecalis* strains.

The results indicate that the methanolic extracts of *A. indica* and *M. oleifera* inhibit 50% of cellular viability (CC_50_) at a concentration of 70 *µ*g/ml. Furthermore, none of the extracts produced any adverse effects on the MDCK cellular lines at low concentrations. We observed an indirect correlation between the concentration and cellular viability (Figure 1).

## 4. Discussion

There are many different types of endodontic infections described. One of them is the persistent root canal infections for which the highly antibiotic-resistant *Enterococcus faecalis* is the main etiologic agent, even amongst patients previously treated for this condition [2]. The pathogenicity of these bacteria includes its capacity to penetrate dentinal tubules, its lack of need of other bacteria to survive, its ability to form biofilms in anaerobic and nutrient lacking conditions, and its resistance to acids and alkalis [5, 6, 12]. It is therefore necessary to study new antibacterial agents specific for this bacteria in order to achieve endodontic treatments with a higher success rate and a better prognosis for the tooth.

As a result of the well diffusion method, we were able to determine that the methanolic extracts for *A. indica* and *M. oleifera* had an antibacterial effect against *E. faecalis* during the first 24 and 48 hours. According to Olson and Fahey, the antibacterial effect of *M. oleifera* is due to the chemical compound 4-(4'-*O*-acetyl-*α*-*L*-rhamnopyranosyloxy)-benzylisothiocyanate, whose mechanism of action involves inhibition of essential cellular membrane enzymes [24, 25]. Moreover, Lakshmi et al. mentioned that the active compound responsible for the antibacterial efficacy of *A. indica* is azadirachtin, a cellular membrane synthesis inhibitor [16, 26].

We also found the MIC of the extracts and concluded that no antibacterial effect was evidenced at low concentrations. In 2013, Reyes and Fernández found the MIC of the foliar extract of *A. indica* against *Staphylococcus aureus* to be 35 *µ*g/ml [27]. Muhammad et al. also evaluated the MIC of an aqueous extract of *M. oleifera* against *S. aureus* and found it to be 6.25 *µ*g/ml. This difference could be explained, given the fact that the process of maceration was 3 times the present study, extracting a greater quantity of chemical compounds of *M*. *oleifera* [28].

The minimum bactericidal concentration for the methanolic extract of *A*. indica was 25 *µ*g/ml and 75 *µ*g/ml for *M. oleifera* which leads us to conclude that at lower concentrations, *A. indica* has a greater capacity to cause cellular lysis of the bacteria in comparison with *M. oleifera.*

The methanolic extracts of *Azadirachta indica* (neem) and *Moringa oleifera* (moringa) did not show any cytotoxicity against the cellular line of MDCK under low concentrations. Jung evaluated the cytotoxicity of the extract of *M. oleifera* against a cellular line Cos-7 and demonstrated a lack of cytotoxicity even with concentrations of 600 mg/ml [20]. In 2015, Jumba et al. evaluated the cytotoxicity of the methanolic extract of *A. indica* against the cellular line Vero-E6 and determined a CC_50_ of 149 *µ*g/ml. Despite the difference in these results, the study concludes that the extract stays within a manageable parameter [29].

Additionally, an *in vivo* study with rats showed that none of the subjects suffered any ailment after treatment with *A. indica extract.*

In conclusion, according to this study, *A. indica* and *M. oleifera* demonstrated an antibacterial effect against *E. faecalis* without any toxicity using low concentration. Therefore, they could be considered as alternative antimicrobial agents to use in the root canal therapy field in Odontology. More research is required regarding the cytotoxicity of each of its active components and also its adverse reactions.

The values that were obtained from this study show a direct correlation between the time of exposure to the extract its antibacterial effect. These results are important to continue a line of investigation in relation to fitotherapy applied to Odontology. The purpose of studies in this field is to promote the future creation of treatments based on these natural herbs considering that their use in radicular conduct treatment would lead to a better prognosis.

## Figures and Tables

**Table 1:** *In vitro* antibacterial effect of 2% chlorhexidine and the methanolic extracts of *Moringa oleifera* and *Azadirachta indica* against *Enterococcus faecalis* during the first 24 and 48 hours.

Time	Extracts	Growth inhibition zone (mm)					
		Mean	Standard deviation	Median	Minimum	Maximum	*p* ^*∗*^
24 hours	*Azadirachta indica*	33.16	0.75	33.00	32.00	34.00	<0.01
	*Moringa oleifera*	35.50	1.05	35.50	34.00	37.00	
	Chlorhexidine	19.16	1.33	20.00	17.00	20.00	

48 hours	*Azadirachta indica*	39.83	3.37	42.00	35.00	42.00	<0.01
	*Moringa oleifera*	44.83	0.98	44.50	44.00	46.00	
	Chlorhexidine	27.00	1.09	27.00	26.00	29.00	

^*∗*^Kruskal–Wallis test.

**Table 2:** Bacteriostatic (MIC) and bactericidal (MBC) of 2% chlorhexidine and methanolic extracts of *Moringa oleifera* and *Azadirachta indica* against *Enterococcus faecalis* strains.

Concentrations (*µ*g/ml)	Study groups		
	*Azadirachta indica*	*Moringa oleifera*	Chlorhexidine
100	—	—	—
75	MIC	MIC/MBC	—
50	—	—	—
25	MBC	—	—
12.5	—	—	MIC
6.25	—	—	—
3.125	—	—	
1.56	—	—	MBC

## Data Availability

The data used to support the findings of this study are available from the corresponding author upon request.
